# Glyoxalase-1 overexpression partially prevents diabetes-induced impaired arteriogenesis in a rat hindlimb ligation model

**DOI:** 10.1007/s10719-016-9681-3

**Published:** 2016-06-13

**Authors:** Olaf Brouwers, Liang Yu, Petra Niessen, Jos Slenter, Karolien Jaspers, Allard Wagenaar, Mark Post, Toshio Miyata, Walter Backes, Coen Stehouwer, Maya Huijberts, Casper Schalkwijk

**Affiliations:** 1 Department of Internal Medicine, Laboratory for Metabolism and Vascular Medicine, Maastricht University Medical Center, P.O. Box 5800, 6202 AZ Maastricht, The Netherlands; 2 Department of Radiology, Maastricht University Medical Centre, Universiteitssingel 50, Maastricht, the Netherlands; 3 Department of Physiology, Maastricht University Medical Centre, Universiteitssingel 50, Maastricht, the Netherlands; 4 Centre of Translational and Advanced Research, Tohoku University, Sendai, Miyagi Prefecture 980-8577 Japan

**Keywords:** Glyoxalase-I, Diabetes, Arteriogenesis, Magnetic resonance angiography, Advanced glycation end-products

## Abstract

We hypothesize that diabetes-induced impaired collateral formation after a hindlimb ligation in rats is in part caused by intracellular glycation and that overexpression of glyoxalase-I (GLO-I), *i.e*. the major detoxifying enzyme for advanced-glycation-endproduct (AGE) precursors, can prevent this. Wild-type and GLO-I transgenic rats with or without diabetes (induced by 55 mg/kg streptozotocin) were subjected to ligation of the right femoral artery. Laser Doppler perfusion imaging showed a significantly decreased blood perfusion recovery after 6 days in the diabetic animals compared with control animals, without any effect of *Glo1* overexpression. *In vivo* time-of-flight magnetic resonance angiography at 7-Tesla showed a significant decrease in the number and volume of collaterals in the wild-type diabetic animals compared with the control animals. *Glo1* overexpression partially prevented this decrease in the diabetic animals. Diabetes-induced impairment of arteriogenic adaptation can be partially rescued by overexpressing of GLO-I, indicating a role of AGEs in diabetes-induced impaired collateral formation.

## Introduction

Arterial occlusive lesions caused by atherosclerosis lead to cardiovascular and peripheral arterial diseases and are the leading causes of death in modern society [1]. In the presence of arterial occlusions, the fate of the affected organ is not only related to the severity of the occlusion, but also by the capability of the developing collateral vessel system to compensate blood perfusion loss. This development of new vessels is significantly reduced in patients with diabetes [2]. Therefore, patients with diabetes suffer from both more arterial occlusion and less compensatory collateral capacity, leading to more foot ulcerations and lower extremity amputations than in non-diabetic patients [1].

Hyperglycaemia is the initiating cause of diabetic tissue damage, and there are several mechanisms that mediate these effects [3]. One of the possible mechanisms is the production of advanced glycation endproducts (AGE) or their reactive precursors. AGEs are formed from reducing sugars reacting non-enzymatically with amino groups in proteins through a so-called Maillard reaction, resulting in dysfunctional modified-products. The major sources of intracellular glycation are the glycolytic intermediates, methylglyoxal (MGO) and glyoxal (GO). Under physiological circumstances these reactive oxo-aldehydes can be efficiently detoxified by the glyoxalase system, in which the enzyme glyoxalase-I (GLO-I) is the rate-limiting step [4].

Previous research showed that elevated levels of MGO in cultured endothelial cells cause cell detachment, anoikis and impaired tube formation [5], which could be prevented by GLO-I overexpression [6]. Furthermore, Liu *et al*. have extensively shown *in vitro* that MGO also impairs endothelial cell viability, migration, tube formation, autophagy, and angiogenesis in *ex vivo* aortic rings, which could be rescued by overexpression of *Glo1* [7]. However, despite these *in vitro* studies and the recent finding that *Glo1* overexpression restores ischaemia-induced angiogenesis in diabetic mice as measured by laser Doppler, [8], the *in vivo* effect of GLO-1 overexpression on specifically collateral formation is unknown. We therefore used magnetic resonance angiography in a diabetic *Glo1* overexpressing rat hindlimb ligation model to investigate if diabetes-induced impaired collateral formation could be prevented.

## Materials and methods

### Diabetic hindlimb ischemic model in rats

This study was approved by the Maastricht University animal ethics committee. Wild-type and GLO-I transgenic rats which were non-diabetic (WtC and TgC respectively) or diabetic (WtD and TgD respectively) for a period of 12 weeks (55 mg/kg streptozotocin) were subjected to ligation of the right femoral artery (*n* = 10 per group). The femoral artery was occluded by placing ligations 0.5 cm below the branch of the circumflex femoral artery and just above the bifurcation of the popliteal and saphenous artery. During the ligation procedure, the Laser Doppler perfusion imaging (LDPI) and magnetic resonance angiography (MRA) exams, rats were ventilated with 3 % isoflurane in oxygen. The animals received Temgesic® (Schering-Plough BV, 0.01 mg/kg subcutaneously) as postoperative medication.

### Laser Doppler perfusion imaging

LDPI (Moor Instruments Ltd., Devon, UK) was used to measure hindlimb blood flow after ligation. Before measuring perfusion, animals were anesthetized and placed on a warming pad to ensure constant body temperature. A low-intensity laser light beam (λ = 632.8 nm) scanned the surface of the skin without contact at a standardized working distance. Scan modus was set at 10 ms/pixel and resolution at 256 × 256 pixels. Three scans were completed per time point for each animal for both the ischaemic and non-ischaemic limbs and average perfusion in arbitrary units (flux) was determined separately for each limb. Perfusion in the ischaemic limb was normalized to the contra-lateral non-ischaemic limb to minimize variation due to ambient light and temperature. Baseline perfusion was assessed preoperatively and postoperative immediately after surgery, and after 3- and 6-days recovery. The normalized perfusion was used to calculate percentage of baseline perfusion at the postoperative time points.

### Magnetic resonance angiography and **collateral quantification**

Seven days post ligation the animals were imaged in supine position in a 7.0 Tesla MR system with a birdcage quadrature coil (Bruker Biospin, Ettlingen, Germany) as described earlier [9]. Briefly, the angiography protocol consisted of a multi-slice 2D flow-compensated gradient echo sequence. A flow saturation slab located distally to the imaging plane was applied to suppress venous enhancement.

The number of visible collateral arteries, based on Longland definition [10] was counted in the medical image software application OsiriX (version 3.7) using axial maximum intensity projections in the cranio-caudal direction over a limited range of axial slices. The thickness and location of the slab was adjusted to obtain optimal depiction of the collaterals. In addition, quantification of the collateralization was assessed by signal intensity distribution analysis (for a detailed description, see [9]) The collateral index represents the normalized volume fraction of vessels with a diameter of approximately 0.5 mm and is therefore used as a measure for the volume of collateral arteries.

### Statistics

All values are expressed as mean ± SEM. Statistical differences between groups were tested using one-way ANOVA with a post-hoc Bonferroni correction for the groups of interest. A *p*-value of less than 0.05 was considered statistically significant.

## Results

Fasted glucose levels 12 weeks after STZ were significantly higher in WtD animals (20.0 ± 1.5 mM) compared WtC animals (3.8 ± 0.1 mM), without effect of GLO-I overexpression (3.5 ± 0.1, and 23.9 ± 1.3 mM for TgC and TgD animals, respectively).

**Fig. 1:**
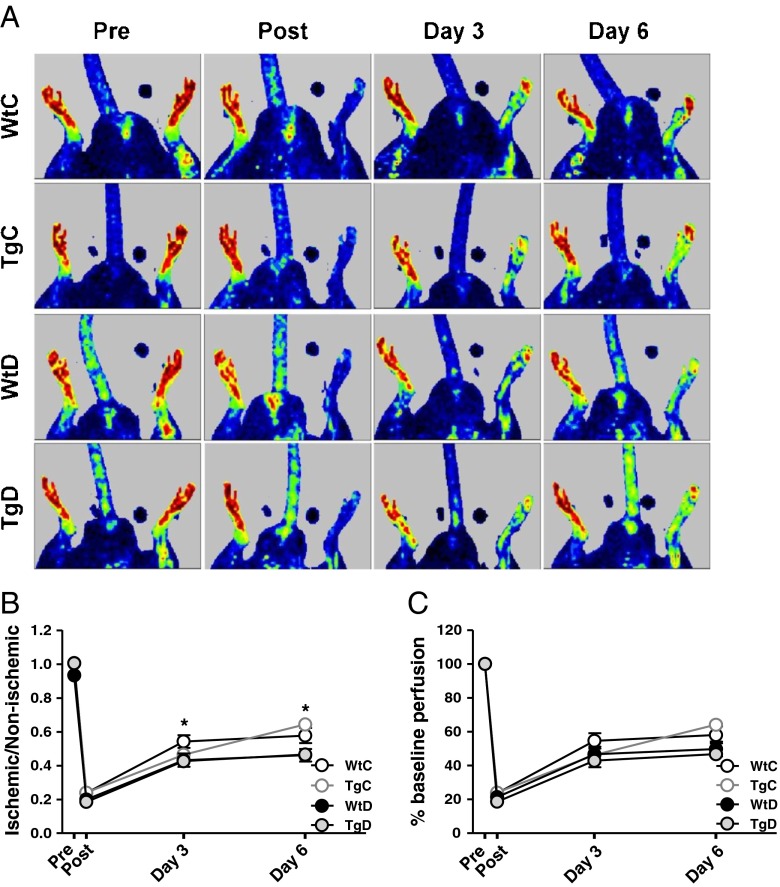
Diabetes impairs recovery of peripheral blood flow after hindlimb ligation without any effects of Glo1 overexpression. Blood perfusion in the paw was measured with LDPI in wild-type control (WtC), transgenic control (TgC), wild-type diabetic (WtD) and transgenic diabetic (TgD) rats before (Pre) and after (Post) ligation of the right femoral artery, and on day 3 and day 6 after ligation **a**. Recovery was quantified by determining the perfusion ratio between the ischaemic and the non-ischaemic paw **b**. or by perfusion percentage with the perfusion before the ligation set on 100 % **c**. * indicates a *p*-value <0.05 when comparing WtC with WtD

LDPI was used to measure the perfusion in the distal area of both the ischaemic and non-ischaemic limbs preoperatively, postoperatively and on postoperative days 3 and 6 (see Fig. 1a). Blood flow is reported for each group as the ratio of blood flow in the ischaemic hindlimb to the non-ischaemic hindlimb (I/NI, see Fig. 1b), and as a percentage of the perfusion recorded at baseline (see Fig. 1c). Immediately postoperatively, blood flow decreased to on average 22 ± 2.2 % of baseline, without any significant differences between the groups, indicating that the hindlimb was effectively and equally rendered ischaemic in all animals. There was a significant decrease in limb blood flow, beginning on day 3 in WtD as compared with and the WtC group for I/NI value. (*p* < 0.05) This difference continued to day 6 (*p* < 0.01), without any effect by GLO-I overexpression.

**Fig. 2:**
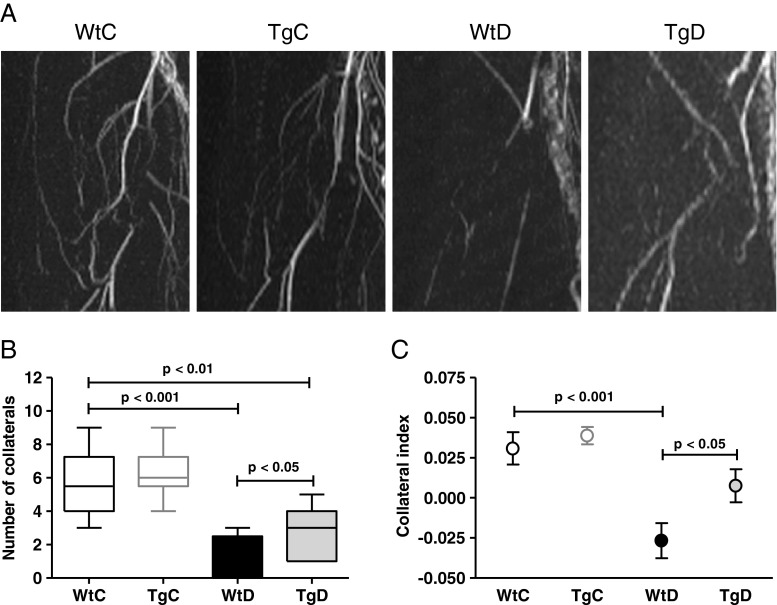
Glo1 overexpression partially prevents diabetes-induced impairment of collateral growth after hindlimb ligation. Collateral growth was imaged with MRA **a**. and the number **b**. and intensity **c**. of the collaterals were quantified with the application software OsiriX

Because laser Doppler measurements are restricted to the superficial skin blood flow distal to the occlusion, we also applied MRA to assess deep arteriogenesis at the actual site of ligation. As measured by MRA, 7 days after ligation (Fig. 2a), the number of collateral arteries was decreased in WtD compared to WtC rats (Fig. 2b). This decrease could be partially prevented by GLO-1 overexpression. In line, the collateral index (*i.e*. the collateral artery volume) (Fig. 2c) showed comparable results between WtD *versus* WtC and also an improvement in the TgD rats.

## Discussion

We showed that diabetic rats with a *Glo1* overexpression displayed significantly more collateral vessels with a higher signal intensity at the site of ligation than their wild-type diabetic littermates.

The mechanisms by which specifically MGO can compromise collateral formation have been extensively investigated *in vitro*. MGO has been shown to modify RGD and GFOGER integrin binding sites of collagen, causing endothelial cell detachment, anoikis, and inhibition of angiogenesis, thereby theoretically impairing the remodeling of the collaterals [5]. Furthermore, MGO also compromises the binding of VEGF to VEGFR2 and thereby the angiogenic process [7]. We show, using a state of the art imaging technique, that this MGO-induced collateral damage also occurs *in vivo*.

Despite the beneficial effect of *Glo1* overexpression on collateral growth at the site of ligation as measured by MRA, *Glo1* overexpression did not improve blood flow in the paws of the rats. After 7 days the paws of the rats showed no signs of necrosis and the ligation did not affect movement behavior of the rats in any group. Any differences in functional capacity of the hindlimb can only be addressed with a treadmill exercise test, which we unfortunately did not perform. Furthermore, preservation of tissue blood flow in the distal hypoxic part of the limb is also dependent on the process of angiogenesis, which occurs later during recovery.

In summary, our observations show that overexpression of *Glo1* promotes collateral growth in diabetic rats *in vivo.* Care should be taken when addressing collateral formation with laser Doppler techniques. Our research suggests that overexpression of *Glo1* leads to quenching of oxo-aldehydes and the inhibition of AGE formation as observed in earlier studies [4, 11]. Therefore, increasing the expression of Glo1, as recently demonstrated in an randomized, placebo-controlled crossover clinical trial with trans-resveratrol and hesperetin, [12], can be an important new tool to prevent diabetes-induced impaired arteriogenesis.
